# Multicentric castleman's disease resembling metastatic lung carcinoma. A case report

**DOI:** 10.1002/ccr3.1381

**Published:** 2018-01-22

**Authors:** Alberto Testori, Emanuele Voulaz, Marco Alloisio, Valentina Errico, Umberto Cariboni, Matilde De Simone, Ugo Cioffi

**Affiliations:** ^1^ Deparment of General and Thoracic Surgery Humanitas Research Hospital Rozzano Milan Italy; ^2^ Deparment of Surgery University of Milan Milan Italy

**Keywords:** Castleman's disease, chemotherapy, lobectomy, lymphadenectomy, PET

## Abstract

A 67‐year‐old patient presented for persistent cough. Computed tomography showed right lower lung opacity associated with mediastinal adenopathy. On suspicion of metastatic pulmonary neoplasm, the patient was submitted to right lower lobectomy with lymphadenectomy. Postoperative histopathology led to the diagnosis of multicentric Castleman's disease.

## Introduction

Castleman's disease (CD) is a rare, usually benign lymphoproliferative disorder, most commonly located in the mediastinum as a solitary slow‐growing mass. It was first reported in 1954 by Benjamin Castleman, who described a 40‐year‐old male with a mediastinal mass characterized histologically by lymph node hyperplasia and follicles with small, hyalinized foci 1, 2.

The histological variants of CD are: hyaline vascular type, plasma cell type, and mixed cell type (rare), each having distinctive histopathological and radiological characteristics 1, 3. Although CD is localized in the majority of cases, a proliferative type multicentric form affecting more lymph nodes in more than one region has been recognized, both clinically and radiologically 4.

The prevalence of CD is unknown, but it is calculated in <1/100,000. The localized form is the most frequent (more than 400 cases described). The multicentric variant, more rare, may be associated with HIV and human herpesvirus‐8 (HHV‐8) infections and can occur at any age 3, 5.

Localized CD is asymptomatic in 51% of cases and is often discovered incidentally 1. It can cause pain in the chest or abdomen, when the mass lesion is large (6 cm in diameter on average, with a range of 1–12 cm). The most affected sites are, in decreasing order of frequency, chest, neck, abdomen 5. The multicentric variant is always symptomatic: fever, night sweats, and weight loss, as well as generalized lymphadenopathy and hepatosplenomegaly and POEMS syndrome (polyneuropathy, organomegaly, endocrinopathy, monoclonal protein, skin changes) are the most frequent symptoms. More severe symptoms include inflammatory vascular leak syndrome, bronchiolitis obliterans, glomerulonephritis, cerebellitis, myasthenia gravis, and pemphigus 2, 5, 6. We report a case of multicentric Castleman's disease presenting as pulmonary opacity in the right lower lobe plus mediastinal lymphadenopathy mimicking a metastatic pulmonary neoplasm.

## Case History

**Figure 1:**
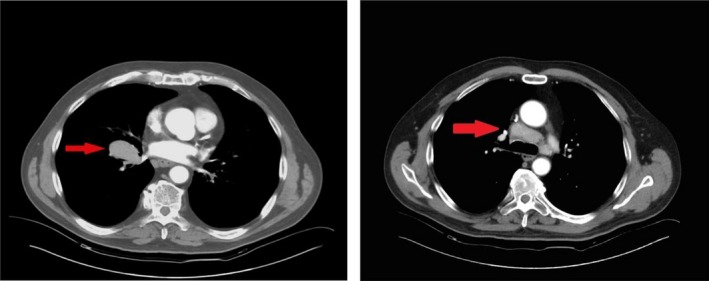
left panel: CT scan showing opacity of the right lower lobe; right panel: CT scan showing precarenal, paratracheal, subcarinal nodes N2.

A 67‐year‐old patient came to our attention for persistent cough not responsive to medical treatment. Chest computed tomography (CT) showed a pulmonary mass located in the right lower lobe with mediastinal adenopathy (Fig 1) both characterized by pathological increased uptake of 18 F‐fluorodeoxyglucose at positron emission tomography (FDG‐PET). Subsequently, the patient underwent a bronchoscopy that showed modest flaring of the carina between the upper lobar bronchus and the intermediate bronchus, with no signs of infiltration. The patient was HIV negative.

Suspecting a metastatic pulmonary carcinoma, the patient was submitted to diagnostic video mediastinoscopy with biopsy of the lymph nodes of the station 4R, 4L, negative for nodal metastasis.

Considering that the histological result was not detrimental, it was decided to perform an explorative thoracotomy that revealed voluminous hilar, mediastinal and interscissural adenopathies. Fine needle aspiration biopsy showed the presence of nonsmall cell in the context of lymphoid tissue. Because it does not rule out the possibility of lymphoproliferative disease or metastatic lung carcinoma, it was decided to perform a right lower lobectomy with extended lymphadenectomy. The postoperative course was uneventful.

**Figure 2:**
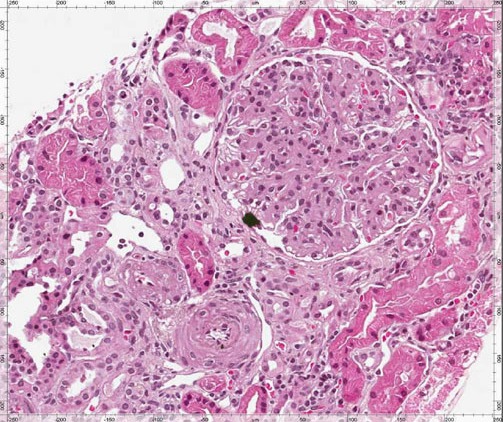
Diffuse mesangial hypercellularity, extracellular matrix augmentation, tubulointerstitial atrophy, rares tubules with cylinders (PAS 10×).

Histology of the resected specimen showed lymph nodes characterized by diffused interfollicular and medullary plasmacytosis. The light chain is either *kappa* or *lambda* in type with mature and polyclonal cells. Follicular B cells resided in lymphoid follicles containing germinal centers, with partial sclerosis. Atypical and large lymphoid cells (HHV‐8+) in some lymph node stations showed morphophenotypic characteristics referable to plasma cell neoplasm. These neoplastic elements expressed the immunophenotypic profile CD20+, MUM1+, HHV‐8+, Bcl2+, CD138+ (weary), Kappa+, Lambda‐, CLA+‐, PAX5‐, CD79a‐, Bcl6‐, EMA‐, LMP1‐, CD10‐, CD30‐. The proliferation index of Ki67 was 80%. This morphophenotypic profile is typical of CD angiofollicular multicentric lymph node hyperplasia) (Fig 2). The definitive histological diagnosis was plasmablastic variant CD.

Following multidisciplinary consultation between surgeons, hematologists, and cardiologist, it was decided to carry out treatment with chemotherapy (liposomal doxorubicin associated with rituximab for six cycles).

At the end of the chemotherapy, the patient was submitted to examinations with contrast medium total body CT scan and PET that showed complete remission of the disease. After 5 years of follow‐up (with blood tests, chest x‐ray, and abdominal ultrasound), the patient is in good health condition without any relapse of the disease.

## Discussion

CD is a rare atypical lymphoproliferative disorder that can be easily misdiagnosed. It can occur anywhere in lymph nodes, most commonly in the mediastinum 7.

The value of the PET/CT in the preoperative diagnosis is low because it is difficult to differentiate malignant from benign diseases such usually as CD 8. The diagnosis is very difficult in these patients, and a lymphnode biopsy is usually needed for the definitive diagnosis and treatment.

If the disease is localized, a complete surgical excision is required, without further therapy (the 10‐year overall survival rates are over 95%). Multicentric Castleman's disease (MCD) affects more than one group of lymph nodes. It can also affect other organs containing lymphoid tissue such as in our case (lung). This form sometimes occurs in people infected with human immunodeficiency virus (HIV). So HIV and HHV‐8 must be tested, and immunostaining is also necessary to exclude the presence of a lymphoma.

Polychemotherapies such as CHOP (cyclophosphamide, doxorubicin, vincristine, and prednisone) are good for healing with a median overall survival of 38 months, but they are highly toxic. Alpha interferon, alone or in combination with vinblastine or etoposide, has proved beneficial for symptom relief. Retinoic acid has been successfully tested in a number of patients; rituximab was used in patients HIV+ and HHV+, with very promising results; cidofovir or ganciclovir has been used successfully in some patients HHV‐8+ 5.

Our case appears in some ways outstanding: because the clinical and radiological signs oriented for lung cancer, only definitive histological examination allowed us to make true differential diagnosis between CD and lung carcinoma 8, 9.

## Conclusion

Castleman's disease can be easily misdiagnosed as infections or cancer. It can be argued that the diagnosis of CD should be considered only after having excluded all the most common causes of lymphadenopathy and must be supported by histological examination.

Once an exact diagnosis is made, complete surgical excision is the treatment of choice in patients affected by hyaline vascular type reserving immunosuppressive therapy and radiotherapy for inoperable or multicentric forms.

## Competing Interest

None of the contributing authors have any conflict of interest, including specific financial interests or relationships and affiliations relevant to the subject matter or materials discussed in the manuscript.

## Consent for Publication

Written informed consent was obtained from the patient for publication of this case report and any accompanying images. A copy of the written consent is available on request.

## Authorship

AT: performed the operation, carried out the study, and revised the manuscript; EV: performed the operation, carried out the study, and revised the manuscript; MA: performed the operation, carried out the study, and revised the manuscript; VE: collected information on the patient; UC: collected information on the patients; MDS: helped in drafting the manuscript and revised the contents of the discussion of the manuscript; UC: carried out the concept and the design of the study and revised the manuscript.
